# An educational programme for nursing college staff and students during a MERS- coronavirus outbreak in Saudi Arabia

**DOI:** 10.1186/s12912-015-0065-y

**Published:** 2015-04-16

**Authors:** Bridget V Stirling, Jennie Harmston, Hana Alsobayel

**Affiliations:** College of Nursing, Princess Nourah University, Riyadh, Saudi Arabia

## Abstract

**Background:**

The Middle Eastern Respiratory Syndrome Coronavirus is a serious and emerging issue in Saudi Arabia and the world. A response was required to reduce possible disease transmission between the hospital and university. College of Nursing academic staff developed a programme in response to the educational and emotional needs of participants.

**Methods:**

A MERS-CoV Task Force responded to the rapidly unfolding epidemic. The aim was to find out what nursing staff and nursing students in the college knew about MERS- CoV. While most gaps in knowledge were addressed after an intense information seminar, other learning needs were identified and responded to.

The Task Force developed mandatory information sessions for all nursing faculty, students and staff. All staff were informed by email, letters and posters. There are 28 faculty staff, 84 support staff and 480 students in the College of Nursing.

The information settings all took place within the College of Nursing, Princess Nourah University, Kingdom of Saudi Arabia.

Questionnaires were given to faculty, students and staff to understand their baseline knowledge. After the sessions, faculty, students and staff were asked about what was learned through the sessions, and what educational needs still needed to be addressed.

Approval was sought and received by the Ethics Committee for the College of Nursing. Participants completed informed consent forms and the voluntary nature of the study was explained.

**Results:**

The total number of people attending the education sessions was133, including 65 students. 18 faculty members attended and 57 support staff. Data was gathered on gaps in participant knowledge and a plan was developed to address the gaps. Policies were established around student participation in clinical and return to work practices for staff with any symptoms.

**Conclusion:**

In hospitals there is above average risk for exposure to infectious diseases. Student nurses travel between hospital and university, with the capacity to act as a conduit of pathogens to large, susceptible populations. Nursing colleges must respond thoroughly to protect students and staff and prevent spread of disease into the university community in the midst of an epidemic.

## Background

MERS-CoV (Middle Eastern Respiratory Syndrome Coronavirus) is a serious and emerging issue in Saudi Arabia and indeed the world today. How governments, hospitals, universities and communities respond will determine to what extent the epidemic becomes widespread within the country, region and the world. Princess Nourah University is the world’s largest all-female university. It is a well-resourced institution, situated on a new campus, and home to more than 40,000 students and approximately 5,000 faculty and staff. Operating in the new campus for less than a decade, the university is working hard to establish itself and to have all departments functioning effectively. This paper is a description of the programme initiated by the College of Nursing in response to an upsurge in cases of MERS-CoV in Riyadh.

Riyadh is the capital city of the Kingdom of Saudi Arabia, and home to 30 million people (one- third are expatriates) [1]. In 2012, Saudi Arabia became aware of the first case of a novel coronavirus (CoV), which later became known as MERS for Middle Eastern Respiratory Syndrome [2]. The first case of MERS-CoV was identified in a patient from the west coast of Saudi Arabia in 2012, after the patient presented with pneumonia and renal failure [3]. The next case was identified in a Middle-Eastern man being treated in a hospital in the United Kingdom [4].

Between the time that it was identified and May 15, 2014, the World Health Organization [5] reported 572 laboratory confirmed infections, with 173 deaths. The highest proportion of confirmed cases and death were (and are currently) in Saudi Arabia. As of yet, the period of time during the spring of 2014 represented the highest peak in cases, throughout the epidemics first two years. Table 1 outlines the number of confirmed cases of MERS-COV up to this date.

**Table 1:** **Number of confirmed cases of MERS-CoV, number of deaths and health workers affected in the Middle East up to the Intervention Period**

**Month and year**	**Number of laboratory confirmed cases (accumulative)**	**Number of deaths (accumulative)**	**Healthcare workers (accumulative)**
30 November 2012	9	5	Not available
25 April 2013	17	7	10 probable cases
8 May 2013	30	14	2
17 May 2013	40	20	2
31 May 2013	50	30	2
20 June 2013	64 (2 children)	38	2
9 July 2013	80 (6 children)	45	4
13 August 2013	94	47	8
20 September 2013	130	58	13
20 January 2014	178	76	32
27 March 2014	206	86	32
24 April 2014	254	93	32
9 May 2014	536	145	58

Source: WHO, Global Alerts and Responses, Coronavirus infections.

As of March 26, 2015, MERS-CoV is confirmed to have infected 1,090 people worldwide, killing 412 of them [6]. This results in a case-fatality rate of more than 37%. According to the WHO report of December 2, 2014 [7], during the month of November 2014, there were 18 new cases, half of which were identified in Riyadh. Of those from Riyadh, 4 had no known risk factors, 3 were people who had visited hospitals, one was a health care worker, and one was a known contact of a previous case. Clearly, hospitals are an important environment to consider when aiming to reduce the spread of MERS-CoV. This also highlights the fact that the university students and staff members are also at risk.

Responding quickly to an epidemic is important in order to facilitate disease control and to protect students, staff and the community from fear and disruption of work. In the case of the surge of cases in this epidemic, the College of Nursing quickly responded to the needs of students and staff by both by providing them with information and listening to their concerns. The College of Nursing also developed policies to protect the university and hospital communities during the time of the epidemic. The use of a small task force was imperative as large committees sometimes take a long time to form, meet and agree on a way forward.

While there was some awareness and concern about the MERS-CoV, unified activities were limited until the rate of diagnosis and death from the virus began to be regularly reported in the media. In April and May of 2014, the Faculty of Nursing underwent a programme to respond to the educational and emotional needs of Faculty, students’ families, and support staff. The response was timely and based on the most current epidemiological data. The College of Nursing takes seriously its duty of care to the student nurses, faculty and staff in clinical placements. So close to the end of semester clinical placements were ceased for students in the pre-internship phase. Increased hours in clinical laboratories were undertaken to ensure no loss of time for students to practice skills.

The WHO guidance [2] states that infection prevention and control measures are critical to prevent the possible spread of MERS-CoV in health care facilities.

Hospitals that provide for patients suspected or confirmed to be infected with MERS- CoV should take appropriate measures to decrease the risk of transmission of the virus from an infected patient to other patients, health-care workers and visitors.

Health care workers should be educated, trained and encouraged to practice skills that aid in infection prevention and control. It is not always possible to identify patients with MERS-CoV early because some have mild or unusual symptoms. For this reason, it is important that health-care workers apply standard precautions consistently with all patients – regardless of their diagnosis – in all work practices all the time.

Advice given by the Ministry of Health, Kingdom of Saudi Arabia includes that people should avoid close contact with animals when visiting farms (especially barn areas) where the virus is known to be potentially circulating. This is difficult as camels and farms are an integral part of the Saudi culture.

## Methods

The response to the upsurge in cases of MERS-CoV required the formation of a task force that would take on a six-pronged approach to meeting the needs of faculty, students and staff. The following plan was enacted:

1. Establishing a helpline to inform anxious nursing students their families about risk and offer support;
2. Developing policies to direct the response to the infection, emergency planning, attendance/absence and return to work;
3. Providing additional resources (WHO correct hand washing diagrams) to highlight the need for frequent hand washing and hand sanitization;
4. Initiating information sessions offered to all faculty, nursing students and support staff (these were mandatory). Key information was made available on the WHO website which highlighted epidemiological data and infection control information;
5. Administering pre and post questionnaires to the Faculty, students and support staff about their current knowledge and feelings about the virus, with a follow up email with further information; and
6. Displaying visual imagery from the Saudi Arabian Ministry of Health in prominent places.

The mandate of the Task Force was to meet the educational and emotional needs of the faculty, students and staff at the College of Nursing. This was initiated in response to concerns brought to the senior faculty by people inside and outside the university. The Task Force decided to offer mandatory information sessions for all faculty and staff, using information that had been confirmed from the published literature and reputable national and international websites.

Task force members consisted of two senior faculty (one from the UK- JH and the other from Canada, BVS) and two junior Saudi faculty members. The two senior faculty had experience in controlling respiratory infectious disease outbreaks in the past. Two administration staff members were called on to translate information into Arabic and provide oral translation during the sessions. The questionnaires were back- translated into English to ensure accuracy. The senior faculty members completed the literature search, highlighted and summarized information from the KSA Ministry of Health website and documents, and prepared all of the learning materials. They organized the training sessions, drafted the policies for approval by the College Council, and communicated with Faculty and staff. Communication was made with the Interns and the Hospital Nurse Educators (through the Internship Unit) and communication with parents and concerned students was done through the Student Services Department.

Due to the urgency of the situation and the senior faculty’s desire to be responsive, the pre and post questionnaire was developed by the Task Force. The purpose was to capture existing knowledge and any changes of current behaviour (pre-test) and in the post questionnaire acquired knowledge and intended changes in their behaviour.

Questions were written by the committee to capture the participants’ awareness of the most important facts, identify gaps in the participants’ knowledge and to guide the follow-up activities. Table 2 outlines the different content provided to each group of participants.

**Table 2:** **What was presented at the educational sessions**

	**Students and faculty (English)**	**Support staff (English and Arabic)**	**Intern students (English)**
What is an epidemic?	Included	Included	Included
What we know and what we are unsure of	Included	Included at a basic level	Included
Signs and symptoms of MERS-CoV	Included	Included	Included
Response from the Ministry of Health to the epidemic	Included	Included	Included
How does the epidemic change how we act as a university community?	Included	Not included	Included
What does epidemic mean for nurses?	Included	Not included	Included
Standard precautions	Included	Not included	Included at an advanced level
Lessons learned from other epidemics	Included	Included	Included
Patient and population profiles	Included	Included at a basic level	Included at an advanced level
Where to access further data (websites, call centers, MERS-CoV committee members)	Included	Included	Included
Response of the Ministry of Health to recent increase in cases of MERS- CoV	Included	Included	Included
Recommendations as to how to act in the hospital setting	Included at a basic level	Not included	Included at an advanced level

The main messages that the MERS-CoV task force at the Princess Nourah University wanted to get across was the following:

- Take the epidemic seriously, but do not be afraid
- Use only accurate information to guide your practice from reputable sources
- Share that accurate information with your family and community
- Follow hospital and university infection control policies and protocol
- Stay home if you are sick
- Use droplet precautions with all patients of suspected MERS-CoV or any respiratory illness, and to use airborne precautions when you are involved in any treatment or activity that aerosolizes the virus.

The Student Services department fielded all calls and concerns, responding to student and family emotional and information needs regarding exposure to the virus in clinical settings. During the information sessions, students and staff were reminded of their role as health educators and the need to continually update themselves with information from credible sources (especially the World Health Organization and the Saudi Arabian Ministry of Health), and inform their family and community as needed.

Another important aspect of infection control during an epidemic is the movement of large groups of people between areas of high concentration of infected people to areas of high concentration of susceptible and uninfected people. Movement between hospitals serving MERS-CoV patients and the universities therefore should be limited. Where a respiratory virus outbreak in hospital occurs, hospital services may be under increased pressure and the risk of infection to the general community is greater. Therefore a rapid and effective containment of hospital staff including students should be a priority [8]. It was decided while the cases in the city were disseminated over all of the hospitals, to remove our students for the last week of clinical work and bring them back to the clinical skills lab for more practice with Personal Protective Equipment (PPE) and infection control procedures. Since then, the Ministry of Health has concentrated the confirmed cases of MERS-CoV into designated hospitals. When students returned to clinical practice in the Fall, they worked in all hospitals, except those designated for MERS- CoV and not with patients who are confirmed or probable cases of MERS-CoV.

Intern students are in their final year of study and do not go back and forth between the hospital and the university. They stay within one hospital for a year, working in different departments. It was decided that the Interns should continue their training at the hospitals. Contact was made with each hospital to establish what protection was being provided (PPE) and also what training was being offered to the Intern students. The Interns were invited to attended a workshop to inform them on the latest clinical advice and to remind them of infection control precautions. Students were able to discuss their concerns and were advised appropriately.

Additional resources were provided with constant reminders to students and staff to increase hand sanitization and hand washing. Additional materials for the clinical labs were purchased to ensure correct infection control measures would be taught.

The Task Force sought research ethics approval from the College of Nursing the Ethics Committee and developed informed consent procedures and forms. Participants completed informed consent forms (which were in both Arabic and English) and the voluntary nature of the study was explained.

Prior to start of the session, a pre-test questionnaire was given to all attendees. Information about their existing knowledge was asked, if they had experienced MERS-CoV in the hospital setting and some information about how they were prepared within the clinical setting. During the sessions, students and faculty were presented by means of a lecture on what the virus is, how it spreads and how to protect themselves in the hospital setting, the university setting and the hospital.

Information was clearly presented about who the virus is affecting, the location and spread of the virus and confirmed data. The aim was to increase clarity about what we do know and what we do not know in order to minimize the risk of inaccurate information being spread. The lecture was supported by visual images and messages on PowerPoint slides, as well as a question and answer period. It is vitally important that nursing students understand under which conditions the virus might aerosolize in the hospital setting.

During certain clinical activities and treatments, the virus might aerosolize, thereby becoming spread by the airborne route, rather than the droplet method. Past studies looking at the SARS CoV showed that healthcare workers are more at risk of becoming infected with that coronavirus while participating in certain nursing procedures [9]. For example, the pooled odds ratio for risk of transmission for healthcare workers exposed to suction before intubation is 3.5 (0.5, 24.6). Healthcare workers exposed to tracheal intubation had a 6.6 OR pooled (4.1- 10.6).

For the support staff working at the College of Nursing, another information session was developed, with a lecture and PowerPoint slides translated into the Arabic language. The focus of these sessions was on understanding what the virus was and how to protect themselves in the university environment and the community.

The Task Force created a handout for the students with relevant information developed from the literature. This handout was created in the English language. The students learn all subjects in English, so they have at least a basic understanding of the English language. An Arabic version of the handout was also developed for support staff members (administration, security etc.) who might have limited ability to understand English.

In order to understand baseline knowledge of students and staff, questionnaires were distributed before each session. The questionnaires were voluntary. All people who participated in the questionnaires signed or acknowledged informed consent as per the College of Nursing’s Ethics approval policy. All questionnaires were written in English for students and faculty. The support staff completed questionnaires in the Arabic language.

Following each session, students and staff were given follow-up questionnaires to establish what was learned through the information sessions. All participants were also asked what educational needs still needed to be addressed. A follow-up e-mail to all staff, students and faculty was prepared to address those further information needs.

Another job of the Task Force was to ensure that all resources from the Saudi Arabian Ministry of Health were being utilized to their fullest. These included making sure that posters were prominently displayed in both Arabic and English, and ensuring that videos made by the Ministry of Health were played for all of the students to hear. Faculty were encouraged to emphasize the use of Ministry of Health MERS-CoV telephone hotlines. Clinical staff reinforced hand washing procedures and drew attention to the newly installed hand sanitizer dispensers. Infection control methods for respiratory virus are well studied in the scientific literature [10,11].

## Results

The total number of people attending the education sessions was 140. This included 65 students from 2nd, 4th and 7th semester. 18 faculty members attended and 57 support staff. While the sessions were mandatory, not all staff, students and faculty attended. Faculty members who did not attend were asked to prepare sessions for their students on MERS-CoV using the material collected by the Task Force or other materials.

Two policies were created to guide the College Executive Committee in making decisions during the epidemic. One policy guided staff on when they should return to work after displaying symptoms during the epidemic. The other established protocol around when students should be pulled from clinical placements.

All students and staff were exposed to the Saudi Arabian Ministry of Health videos, posters, and handouts. 20 posters from the Ministry of Health on MERS-CoV prevention were placed throughout the building in elevators, washrooms, hallways, library, etc.

Students were directed to use the MERS-CoV Ministry of Health hotline if they had any questions, to refer to the World Health Organization’s and the Ministry of Health KSA. It was emphasized that these website have accurate updates. Students were also encouraged to increase the use of hand sanitizer and wash their hands frequently.

Over 200 pamphlets created by the Task Force outlining the pertinent information on MERS- CoV for nursing students and staff were handed out during sessions or distributed through other means (classrooms, student lobby, etc.).

### Gaps in knowledge

The results of the pre and post-test showed that while knowledge about MERS-CoV increased and participants were more able to identify the role of a nurse during an epidemic, gaps still existed. Follow-up activities included playing audio messages throughout the College from the Ministry of Health, putting up new posters in Arabic and English throughout the College, as they became available, and encouraging faculty members to include information about MERS-CoV in their lectures. Follow- up meetings with students in the clinical settings during their internship were also held. Students were encouraged to ask questions of the faculty or to call the Ministry of Health hotline if they had concerns or required more information.

## Discussion

There is very little research on the response of nursing colleges during an infectious disease epidemic. The reason for this may be the intensity of work for faculty during this time and the urgency of the situation. Clearly as epidemics become more widely known and as air travel has made the opportunities for disease to move from one region to the next, more research will need to be done to guide policy-makers and university administration staff in what to do, as they occur.

During this epidemic students and staff regularly visited and worked in clinical settings within local hospitals in Riyadh, some of these sites had laboratory confirmed MERS-CoV patients. Ensuring staff and students were appropriately informed of the risks and their role in using standard precautions to reduce cross infection, was a priority for the Task Force. As the recent cases of MERS-CoV indicate, visitors and health care workers in Riyadh and throughout Saudi Arabia carry a measure of risk by being in the hospital environment. Our students became more resilient against the virus by increased education and awareness, heightened use and skill with personal protective equipment and protective policies.

How health care staff and nursing students act in the hospital is different from how they act at the university and in the community. By nature of the fact that the hospital is necessarily filled with both infectious and highly susceptible people, careful thought must be made to infection control practices. Nursing students must be reminded about the dangers of bringing pathogens home on their shoes, clothes, lab coats and in Saudi Arabia, their head coverings. Students are also reminded about hospital safety policies and use of protective resources (gloves, masks, gowns, etc.), especially when working with a suspected case of any infectious respiratory diseases. Further research on the behaviour of nursing students to their clinical settings during an outbreak or epidemic should be considered.

Attendance is always an issue when creating an ad hoc information session, even when people are highly motivated to attend due to the seriousness of the epidemic. The sessions were made mandatory, on direction from the Dean of the College (HA). Those faculty members who were unable to attend were directed to self-educate on the issue and use the materials to do short sessions on the virus, how it spreads, how to prevent becoming infected and what to do if you become ill.

One concern that emerged from the discussions was whether people should be wearing surgical masks in the university environment. There was a notable increase in the number of students across the campus wearing paper masks, with little evidence of them taking them off or changing them. Indeed at the time about 10% of students were wearing paper surgical masks during class. When asked why they were wearing masks, several students answered that they were “preventing corona”. When asked if they meant that they were sick, they answered that they were not, but they did not want to get corona from other students. At the time, there were rumours on social media sites claiming that the university had several cases of the virus. This rumour was likely untrue as it was never validated by the MOH, the WHO or any credible source. Unfortunately, students were observed touching elevator buttons and then adjusting their mask, removing their masks to cough, and wearing a single paper mask throughout a 6 hour day.

In the age of information and social media, it is difficult sometimes for students and their families to differentiate between rumour and fact. Students, staff, and faculty of nursing colleges are “ambassadors” of the health care system. People in the general community listen to nurses and consider what they say seriously. Further research in to the effectiveness of social media during an outbreak should be considered.

## Limitations

Due to the need for a timely response to the increasing MERS-CoV laboratory confirmed cases in Saudi Arabia and in particular increasing numbers in Riyadh, the questionnaire was not piloted and the questions were not validated. A follow-up study should include a study of validation and if needed, revision. This paper did not consider the implications of epidemic during mass gatherings (for example Mecca or Medina), however the size of the university with 45,000 staff and students is a considerable number although spread over 32 colleges. This may be considered a very large gathering of people. No clinical staff from Riyadh hospitals attended the education sessions nor were they involved in the Task Group.

## Conclusion

In conclusion, the Task Force worked hard to respond to the emotional and information needs of the students, faculty and staff at the College of Nursing. In collaboration with the hospitals, Student Services, the Ministry of Health and others, students and staff were able to have any questions answered, and were equipped to protect themselves in the hospital, university and community in Riyadh.

In hospitals and other environments where nursing students are educated, there is a higher than average risk for exposure to infectious diseases. As student nurses travel week-by-week between hospital and the university, they have the capacity to be a conduit of that pathogen to the large and susceptible population. Schools of health sciences, including nursing must respond quickly and thoroughly to protect their students and staff, to prevent spread of disease into the university community and to become ambassadors of accurate information in the midst of an epidemic.
